# Maternal Serum and Breast Milk Adiponectin: The Association with Infant Adiposity Development

**DOI:** 10.3390/ijerph15061250

**Published:** 2018-06-12

**Authors:** Marhazlina Mohamad, See Ling Loy, Poh Ying Lim, Yu Wang, Kah Leng Soo, Hamid Jan Jan Mohamed

**Affiliations:** 1 School of Nutrition and Dietetics, Faculty of Health Sciences, Universiti Sultan Zainal Abidin, Kuala Nerus, Terengganu 21300, Malaysia; 2 Department of Reproductive Medicine, KK Women’s and Children’s Hospital, Singapore 229899, Singapore; Loy.See.Ling@kkh.com.sg; 3 Department of Community Health, Faculty of Medicine and Health Sciences, Universiti Putra Malaysia, UPM, Serdang 43400, Malaysia; pohying_my@upm.edu.my; 4 Department of Pharmacology and Pharmacy, Li Ka Shing Faculty of Medicine, The University of Hong Kong, Hong Kong, China; yuwanghk@hkucc.hku.hk; 5 Nutrition and Dietetics Programme, School of Health Sciences, Universiti Sains Malaysia, Kubang Kerian, Kelantan 16150, Malaysia; sookl@usm.my

**Keywords:** pregnancy, adipokines, adiponectin, leptin, breast milk, obesity, infant adiposity

## Abstract

The prevalence of childhood obesity is increasing at an alarming rate in Malaysia. Metabolic changes during pregnancy are critical to the development of infant adiposity, due to imbalanced adipokines production. Hence, we aimed to investigate the association of maternal serum and breast milk adipokines with infant adiposity development. The study was conducted from April 2010 until December 2012. A total of 155 healthy pregnant mothers aged 19 to 40 years were recruited during the first and second trimester in Kelantan, Malaysia. Data consisted of maternal sociodemographic details, anthropometry and clinical biochemistry analysis; and the infant’s anthropometry and feeding patterns. Maternal fasting serum and breast milk samples were analysed for adiponectin and leptin levels. Data collection was performed in the second and third trimester of pregnancy, and continued with follow-up visits at birth, two, six, and 12 months postpartum. Multiple linear regression (MLR) analyses were performed to examine the associations between maternal serum and breast milk adiponectin and leptin and infant adiposity development. MLR models showed that, in the first year, as maternal serum and breast milk adiponectin increased, infant weight, BMI-for-age Z scores and abdominal circumference significantly decreased (*p <* 0.05). Maternal serum and/or breast milk adiponectin was associated with first-year infant adiposity development.

## 1. Introduction

Obesity has become a serious concern, with the prevalence of childhood obesity soaring. By 2020 it was estimated that the global prevalence of overweight and obesity in children aged 0–5 years could reach 9.1% [1]. The national prevalence of overweight in children aged under five years, according to the National Health and Morbidity Survey (NHMS) 2015, has increased to 7.6% [2], as compared to 6.4% from the NHMS 2006 [3].

Adipokines are adipocyte-derived secretory factors that are responsible for the regulation of multiple metabolic pathways [4], including the modulation of lipid and glucose metabolism [5,6] and the regulation of energy homeostasis [7,8]. Adiponectin and leptin are among the most potent and most studied adipokines in relation to obesity. Findings from previous studies indicated an inverse relationship of maternal serum adiponectin during pregnancy and breast milk adiponectin with infant adiposity [9,10,11,12]. Meanwhile, maternal serum leptin during pregnancy showed a positive association with infant adiposity [13,14]. The changes in maternal metabolism and adipose tissue deposition were related to the alterations of adiponectin levels throughout pregnancy [15]. Irrespective of body weight, pregnant mothers was shown to have a higher serum leptin level as compared to in the non-pregnant state [16,17], and the level of serum leptin was reported to increase with advancing gestation [18,19,20].

The “developmental origins of chronic adult disease” hypothesis proposed by Barker [21] emphasizes the crucial role of the intrauterine environment in the early development and subsequent lifelong health of the foetus. The hypothesis states that an adverse prenatal environment may influence the physiology and metabolism of the foetus, resulting in “programming”, described as a condition in which the foetus changes the body’s structure, function and metabolism as a way to adapt to a scarce environment. The imbalance of intrauterine maternal adipokines may induce foetal programming, which may permanently change the foetal metabolism and appetite regulation. Consequently, it may lead to childhood obesity and the development of health problems in later life. 

Adipokine levels in serum and breast milk vary across populations, which could be attributable to racial and ethnic disparities [22,23]. Although previous studies have reported the associations of serum adiponectin and leptin during gestation and breast milk adiponectin with infant adiposity, the majority were conducted in a Western setting [11,14]; very few have been done in an Asian setting [24]. With the use of data involving an Asian population from the Universiti Sains Malaysia Pregnancy Cohort Study, we aimed to investigate the associations of maternal serum adiponectin and leptin levels during pregnancy and breast milk adiponectin levels with infant adiposity development during their first year of life.

## 2. Materials and Methods

### 2.1. Study Population and Design

This study was part of the Universiti Sains Malaysia Pregnancy Cohort Study. The study recruited healthy pregnant mothers from two antenatal clinics; (1) Obstetrics and Gynaecology Clinic, Hospital Universiti Sains Malaysia (HUSM) and (2) Klinik Kesihatan Kubang Kerian, Kelantan. For the purpose of this study, a total of 155 pregnant mothers were selected using a convenience sampling technique based on inclusion criteria such as (1) Malaysian; (2) Malay ethnicity; (3) aged 18–40 years old; (4) single pregnancy; (5) gestational age ≤24 weeks (two weeks’ time frame before 26 weeks for availability of fasting blood samples); and (6) live within a 50 km radius of HUSM. The sample size was calculated using the a-priori sample size calculator for multiple regression, version 3.0 [25], based on the formula by Cohen et al. [26] (anticipated effect size = 0.15; power = 0.90). Mothers with preterm delivery and pre-existing pregnancy complications (gestational diabetes mellitus, pregnancy-induced hypertension or preeclampsia) or chronic diseases were excluded from the study due to the discrepancy of adipokines level in mothers with metabolic disorders [27,28]. The response rate of 52.5% was mostly due to the refusal of the mothers to give their blood samples. Prenatal assessment in the second and third trimester of pregnancy was done in the antenatal clinic, while postnatal visitation involved ward visits in Hospital Universiti Sains Malaysia or home visits (for mothers who had given birth in other hospitals, followed by visits at two months, six months and 12 months postpartum). The study was carried out from April 2010 until December 2012. Every pregnant mother who agreed to take part in the study was required to give written informed consent. 

### 2.2. Data Collection

Information regarding maternal sociodemographic characteristics (such as age, education, employment and household income), gravidity and infant’s feeding patterns were obtained through face-to-face interview with the mothers. Infant’s feeding patterns included questions on the types of infant feeding (breast milk, formula milk, plain water, or other beverages), duration of breastfeeding (months) and the age of first introduction to complementary food (months). Based on the data from the interview, breastfeeding patterns were classified into three categories: exclusive breastfeeding, partial breastfeeding and no breastfeeding. According to the WHO [29], an infant is said to be exclusively breastfed when only feeding on breast milk and no other additional food or beverage, not even water, except drops or syrups of vitamins, mineral supplements or medicines. Partial breastfeeding was defined as an infant who fed on breast milk as well as receiving water-based drinks, food-based fluid, semi-solid or solid food or non-human milk. Gestational age was determined based on the last menstrual period (LMP) or ultrasound scan that was performed in the first trimester (more than eight weeks’ gestation) when appropriate. Ultrasound date was only used when the LMP was unknown, or when the LMP date was more than seven days from the ultrasound date. 

Anthropometric measurements were used as an indicator of the nutritional status of mothers and infants. For this purpose maternal anthropometry was measured twice during the prenatal period, at the second and third trimester of pregnancy. During the postpartum period, infants were followed up periodically for anthropometric measurements at birth, two, six and 12 months. All measurement procedures were performed by only one measurer to avoid inter-measurer bias, with the assistance of one recorder who also helped position the mothers and infants during the measuring process. Maternal height was measured at the second trimester, while maternal weight was measured at the second and third trimesters of pregnancy. Maternal pre-pregnancy weight was recorded based on a self-reported method. Total gestational weight gain was computed as the last measured maternal weight during pregnancy minus the maternal self-reported pre-pregnancy weight. Gestational weight gain rate was defined as the maternal weight measured at the second or third trimester minus the maternal self-reported pre-pregnancy weight divided by the weeks of gestation attained at the second or third trimester weight measurement. For infant anthropometric measurements, length, weight, abdominal circumference and skinfold thickness were taken. Infant length and weight were measured using an electronic baby scale with attached measuring rod (seca 334, Hamburg, Germany). Measurement of the abdominal circumference was done using a non-stretchable measuring tape. Infant’s skinfold thicknesses were measured at the age of six and 12 months using a Harpenden Skinfold Caliper (British Indicators, West Sussex, UK). Ponderal index was derived from the infant birth weight in kilograms divided by the infant length in cubic metres (kg/m^3^). Standard measurement procedures and techniques were applied in all anthropometric measurements [30,31]. 

### 2.3. Maternal Serum and Breast Milk Collection

Fasting blood sample was collected at the second and third trimesters of pregnancy. The mother was requested to fast for 10–12 h before the blood collection. A total of 2 mL venous blood was drawn and transferred into a blood collection tube for measurement of the total adiponectin, leptin and other biochemistry analyses. The tube was immediately kept in an icebox. Then, the blood sample was centrifuged using an Eppendorf Centrifuge 5810R (Hamburg, Germany) at 3500 rpm for 10 min at 4 °C. Serum from centrifugation was transferred into the microcentrifuge tube and stored at −80 °C (Thermo Fisher Scientific 702, Waltham, MA, USA) until further analysis. 

Breast milk sample was collected at birth and two months postpartum on random occasions, and both fore milk or hind milk were included in the analysis. A total of 0.5 mL breast milk was collected manually by hand expression; in cases where the mother was unable to hand express the breast milk, an electric breast pump (Medela Mini Electric Breastpump^TM^, Manchester, UK) was used. The breast pump was thoroughly washed with soap and warm water time after each use in order to prevent cross-contamination. Collected breast milk samples were transferred into microcentrifuge tubes and immediately stored in an icebox. They were then stored in a −80 °C freezer (Thermo Fisher Scientific 702) until further analysis. 

### 2.4. Adiponectin and Leptin Analysis

The quantification of adiponectin and leptin in serum and breast milk was performed using the enzyme-linked immunosorbent assay (ELISA) method. The serum and breast milk adiponectin levels were analysed using a Human Adiponectin Immunoassay Kit Cat. No. 31010 (Antibody and Immunoassay Services Center, The University of Hong Kong) and High Sensitive Human Adiponectin Immunoassay Kit for Analysis of Milk Sample Cat. No. 31013 (Antibody and Immunoassay Services Center, The University of Hong Kong). The serum samples were subjected to 1000-fold dilution for the assay. The breast milk samples were centrifuged at 1600× *g* for 20 min at 4 °C, and the fatty layers were removed. This step was repeated three times. Then the milk samples were subjected to a 3-fold dilution for the assay. The levels of serum leptin were determined using the commercial Leptin ELISA Kit Cat. No. 11-LEPHU-E01 (American Laboratory Products Company (ALPCO) Diagnostics, Salem, NH, USA) according to the instructions from the manufacturer. For quality control of the test, assays with intra- and inter-assay coefficients of variability (CV) of less than 10% were accepted. The adiponectin to leptin ratio (ALR) was derived from the maternal serum adiponectin level in relation to the maternal serum leptin level.

### 2.5. Dependent Variables

Variables for infant adiposity include body weight, body mass index-for-age *Z* scores (BAZ) and abdominal circumference at birth, two months, six months and 12 months of age.

### 2.6. Independent Variables

Infant adiposity predictors include serum adiponectin and leptin levels in the second and third trimesters of pregnancy as well as breast milk adiponectin levels at birth and two months postpartum. Adjustments were made by gestational weight gain, gestational age, infant sex, maternal age, pre-pregnancy BMI and breastfeeding patterns. 

### 2.7. Data Analysis

All statistical analyses were conducted using IBM SPSS Statistics, Version 22.0 (IBM, Armonk, New York, NY, USA). Data with normal distribution were presented as the mean (standard deviation), while non-normally distributed data were described with median and interquartile range. Categorical data were presented as a number and percentages. Skewed data were transformed to either log or square root to achieve data normality in order to meet the assumptions for parametric tests. Paired sample t-tests were performed to compare the maternal weight and serum adipokine levels between the second and third trimesters of pregnancy, and to compare breast milk adiponectin levels between 0 and two months postpartum. The differences in infants’ anthropometric measurements by sex and the differences in infant weight by breastfeeding patterns at 12 months of age were analysed using the independent *t*-test. Pearson correlation coefficients were computed to examine the correlation between: (1) gestational age and infants’ anthropometric measurements; and (2) infants’ weaning age and weight at six months and 12 months of age. The statistical significance value was set at *p <* 0.05. 

Simple linear regression was performed to determine the statistically significant potential predictors for infant adiposity. Selected variables with *p <* 0.25 from simple linear regression were included in the linear regression analyses with the stepwise method. Predictors with *p <* 0.05 that explained the models best for infant adiposity in the final multiple linear regression models were adjusted for confounders. Confounders were identified based on previous studies on infant adiposity [11,32,33,34] as well as factors that were considered biologically plausible and by proof of statistical evidence; therefore, different confounders were adjusted for each outcome. Interactions, multicollinearity as well as linear regression assumptions of linearity, normality, and equal variance of residuals were checked before the final models were verified. 

### 2.8. Ethics Approval

The study protocol was approved by the Human Research Ethics Committee of Universiti Sains Malaysia [USMKK/PPP/JEPeM (210.3{03})] and the Medical Research Ethics Committee of the Ministry of Health, Malaysia [(2)dlm.KKM/NIHSEC/08/0804/P10-238]. 

## 3. Results

### 3.1. Maternal Characteristics

Table 1 shows the personal background, sociodemographic characteristics, anthropometric measurements and adipokine levels of the mothers. From a paired sample *t-*test, maternal body weight was significantly different between the second and third trimesters of pregnancy (*p <* 0.001), from 57.53 (SD = 10.58) kg to 64.17 (SD = 10.61) kg. There was no significant change in serum adiponectin levels between the two trimesters of pregnancy (*p* = 0.379). However, serum leptin levels were significantly increased in the third trimester as compared to the second trimester (*p* = 0.019). On the other hand, breast milk adiponectin was observed to decrease two months after giving birth, and differed significantly from the concentrations at birth (*p <* 0.001).

### 3.2. Infant Characteristics

Mean (SD) gestational age at delivery was 38.82 (1.11) weeks. Pearson’s correlation test indicated that infant weight (r = 0.24, *p* = 0.003) and length (r = 0.17, *p* = 0.035) at birth was positively correlated with gestational age (Table 2). An independent *t*-test showed that boy infants were born with a significantly higher birth weight (*p* = 0.043) than girl infants. At two months, six months and 12 months of age, boy infants were found to have a significantly higher mean of all anthropometric measurements (*p <* 0.05) except growth indices (*Z* scores) as compared to their girl counterparts. An independent *t*-test showed that infants who were not breastfed at 12 months of age had a significantly higher mean weight compared to those who breastfed (*p* = 0.002). The mean (SD) infant weaning age was 5.43 (0.97) months. Pearson’s correlation test showed that there was no significant correlation between infant weaning age and weight at 12 months (r = 0.10, *p* = 0.219) of age. 

### 3.3. Associations of Serum and Breast Milk Adipokine Levels with Infant Adiposity

Simple linear regression analysis showed that the maternal serum adiponectin level during pregnancy, the adiponectin to leptin ratio (ALR) at second trimester, and the breast milk adiponectin level at two months postpartum were associated with the first-year infant body weight and BAZ (Table 3 and Table 4). The significant association of maternal serum leptin level was only shown in infant BAZ at two months of age. After confounders adjustment (Table 3), there was a significant linear negative relationship between the serum adiponectin level in the second trimester and the six-month infant body weight (β = −0.59, *p* = 0.002), indicating that the increase in serum adiponectin concentration in the second trimester was significantly associated with lower infant body weight at six months of age. The overall model fit was *R*^2^ = 23.7%. A similar direction of relationship was observed in the serum adiponectin level in the third trimester with infant body weight at birth (β = −0.47, *p* = 0.004, *R*^2^ = 18.1%) and 12 months of age (β = −1.02, *p* = 0.009, *R*^2^ = 23.8%). In adjusted multivariable analysis for infant body weight at two months of age, the level of breast milk adiponectin at two months postpartum (β = −0.54, *p* = 0.003) alone was shown as a predictor, with an overall model fit of *R*^2^ = 30.1%. In the adjusted multivariable analysis for infant BAZ at two months of age (Table 4), breast milk adiponectin level at two months postpartum remained the single predictor (β = −0.79, *p* = 0.008), indicating that there was a decrease in two-month infant BAZ with an increase in the breast milk adiponectin level at two months postpartum. The overall model fit was *R*^2^ = 6.3%. The negative linear associations between serum adiponectin levels in the second and third trimesters and infant BAZ at six months (β = −0.62, *p* = 0.009) and 12 months (β = −1.00, *p* = 0.018) of age became stronger after adjusting for confounders. However, maternal serum leptin in the third trimester and ALR in the second trimester only showed negative linear associations with infant BAZ at birth (β = −0.22, *p* = 0.001 and β = −0.94, *p <* 0.001, respectively). 

Simple linear regression analysis in Table 5 revealed that the levels of maternal serum adiponectin during pregnancy and breast milk adiponectin at two months postpartum were associated with infant abdominal circumference in the first six months of life. After confounder adjustment, there was a significant linear negative relationship between the serum adiponectin level in the third trimester of pregnancy and the infant abdominal circumference at birth (β = −2.52, *p* = 0.009). The overall model fit was *R*^2^ = 8.8%. In the adjusted multivariable analysis for two-month infant abdominal circumference, breast milk adiponectin level at two months postpartum was the only predictor retained in the model (β = −2.34, *p* = 0.003), with an overall model fit of *R*^2^ = 16.4%. The linear negative relationship became stronger between serum adiponectin levels in the second trimester and infant abdominal circumference at six months of age (β = −1.42, *p* = 0.023), and the overall model fit was *R*^2^ = 10.2%. None of the serum adiponectin and leptin levels (as well as ALR) was retained in the multivariable model after adjusting for confounders for infant abdominal circumference at 12 months.

## 4. Discussion

### 4.1. Main Findings

Increased maternal serum adiponectin during pregnancy was associated with decreased infant body weight, BAZ and abdominal circumference during the first year of life. The findings demonstrated the protective effect of maternal serum adiponectin during pregnancy on infant adiposity development in the first year of life. The higher level of maternal breast milk adiponectin at two months postpartum was associated with decreased infant body weight, BAZ and abdominal circumference at two months of age. After confounder adjustment, breast milk adiponectin at two months postpartum remained the only predictor for infant adiposity at two months of age. This suggested the protective effect of breast milk adiponectin on infant adiposity development in the first few months of life. However, there was no association found between maternal breast milk adiponectin at birth and two months with infant adiposity at six and 12 months of age. On the other hand, an increased maternal adiponectin to leptin ratio (ALR) was associated with decreased infant BAZ at birth. As ALR was inversely correlated with the homeostasis model assessment of insulin resistance (HOMA-IR) [35], the increase in ALR highlighted the protective role of adiponectin against insulin resistance and its subsequent effect on decreased infant BAZ at birth. The fact that only healthy pregnant mothers were included in this study might explain the absence of a significant association between ALR with infant weight, BAZ and abdominal circumference at two, six and 12 months postpartum. 

### 4.2. Data Interpretation and Comparison with Previous Studies

#### 4.2.1. Associations of Maternal Serum Adiponectin and Leptin Levels during Pregnancy with Infant Adiposity Development during the First Year of Life

The inverse relationship of maternal serum adiponectin during pregnancy with foetal birth weight was documented in several studies [12,36,37,38]. In contrast, one study reported a positive association [39], while several studies failed to find any association between maternal serum adiponectin and birth weight [24,40]. The discrepancy between these studies and our findings might be attributable to the differences in subjects and the stages of pregnancy studied. 

Apart from that, there might be a significant link between maternal and infant adiposity. A cohort study revealed that infants who were born to overweight and obese mothers had a higher birth weight and a greater increase in weight gain during the first year of age than infants of normal weight mothers [21]. Moreover, a significantly positive association between gestational weight gain and birth size was also reported in previous studies [41,42]. However, considering that the aim of the study was to examine the role of adipokine levels in the development of infant adiposity, the maternal pre-pregnancy BMI and total gestational weight gain were controlled as confounders in this study.

Pregnancy is described as a state of insulin resistance that results from changes in the maternal circulating levels of inflammatory mediators [43] and increased adiposity [44]. During the first trimester, a pregnant mother starts to accumulate adipose tissues, followed by increased insulin resistance and lipolysis in later trimesters [45]. The changes in the hormonal milieu during pregnancy that enhance fat tissue storage may reduce sensitivity to insulin [46]. TNF-α, as a predictor of insulin resistance in pregnancy [47], may suppress the function of peroxisome proliferator-activated receptor in adipocytes, thus inhibiting the secretion of adiponectin [48]. 

As pregnancy progresses, maternal insulin resistance is heightened and the adiponectin level is reduced, which may increase the supply of glucose to the foetus [49]. In addition, increased insulin resistance is shown to suppress the inhibition of lipolysis and amino acid turnover, which leads to elevated free fatty acids and amino acids. Subsequently, the oversupply of nutrients together with increased foetal insulin resistance as a response to intrauterine hyperinsulinism may result in the overgrowth of the foetus [50]. Adiposity and the possible existence of impaired glucose intolerance among the mothers in the current study might be related to the decreased serum adiponectin level. It was demonstrated in this study that mothers with a lower adiponectin level had heavier and larger infants. An intrauterine environment stressed by insulin resistance and molecular intermediates such as adiponectin might have programmed the infants through changes in the metabolic and/or appetite-regulating pathways that would predispose them to develop adiposity in the first year of life [50]. 

Within the first six months after birth, the infant builds more fat mass (fat cells rapidly increase in size), then over the next six months the size of fat cells is slightly decreased. The number of adipocytes only starts to increase from the end of the first year of life [51]. Until six months of age, the infant accumulates fat mass up to 25–30% of the total body weight, while over the second six months the fat-free mass comprises a large percentage of total infant body weight [52]. A study reported that higher intake of protein between nine and 12 months of age was associated with increased BMI among boy infants at the age of six years [53]. Taken together, the inverse relationship between maternal adiponectin and infant adiposity in this study might reflect the protective role of maternal adiponectin on reducing weight gain within the critical period of the first six months of infant life, at which fat mass is actively built up. 

On the other hand, the period in the latter half of the first year is when the infant diet changed with the introduction of complementary food, which was not explored in the current study. That might explain the absence of a significant association between maternal serum adiponectin and infant abdominal circumference at 12 months of age. Yet, it is suggested that the higher maternal adiponectin during pregnancy might slow down the catch-up growth of infants within the first 12 months of life, when prevention of childhood obesity should begin.

#### 4.2.2. Associations of Maternal Breast Milk Adiponectin Levels within Two Months Postpartum with Infant Adiposity Development during the First Year of Life

Thus far, there is a dearth of scientific data regarding the associations between these parameters. A study by Woo et al. [11] demonstrated an inverse association between maternal breast milk adiponectin and infant WAZ and WLZ at 0, one and three months of age, in which the stronger cross-sectional association between breast milk adiponectin and infant weight suggested that the effects may rely on current consumption of breast milk. Since the breast milk samples were only collected at birth and two months postpartum in the current study, the association with infant adiposity at six and 12 months of age could not be finalised. The underlying mechanisms and pathways for such an inverse association remain to be elucidated. 

On the contrary, a study by Weyermann et al. [54] found that mothers with large-for-gestational age (LGA) infants had a significantly higher level of breast milk adiponectin as compared to mothers with adequate-for-gestational age (AGA) or small-for-gestational age (SGA) infants. Bronsky et al. [55] reported that breast milk adiponectin levels throughout the first year postpartum were not significantly associated with infant body weight. The discrepancy in the results among these studies might be attributable to the differences in subjects, stages of study, methods of breast milk sample collection and assay, or breast milk adiponectin pattern throughout the respective studied periods.

Studies with a duration longer than 12 months postpartum also reported controversial findings. Weyermann et al. [33] documented that a higher level of breast milk adiponectin at six weeks postpartum was associated with higher odds of infant overweight at two years of age, particularly among those who were breastfed for at least six months. Yet, its underlying mechanism and pathway were not further explored. Another study also indicated that breast milk adiponectin at six weeks postpartum was positively associated with weight gain and the sum of skin-folds up to the age of two years [56]. Woo et al. [57] found that infants exposed to higher breast milk adiponectin experienced a significant increase in weight-for-age *Z* scores (WAZ) between 12 and 24 months of age as compared to those exposed to lower adiponectin. This finding conflicted with their previous work, which reported an inverse relationship between breast milk adiponectin and infant growth in the first six months of life [11]. However, since WAZ was consistently below the median, and overweight at 24 months of age was uncommon in the latter study, Woo et al. [57] also concluded that the greater weight gain in the latter half of the second year might not reflect the pathology of obesity but rather the positive catch-up growth after a less evident weight gain within six months postpartum. It was suggested that delayed catch-up growth within the first half of the second year represented the protective effect of higher breast milk adiponectin against adiposity, since more fat mass depositions occur during the period. 

In contrast, preliminary results from a prospective follow-up study reported that colostrum adiponectin was inversely associated with children’s BMI at 10 years old [58]. Findings from the study were described with regards to the innate immune system regulation. The alteration in the composition of gut microbiota could influence energy homeostasis, in which glucose is rapidly absorbed and lipid is excessively stored [59]. Thus, it was proposed that the higher colostrum adiponectin among normal-weight children might suppress the proinflammatory response in the infant’s intestines due to the changes in gut microbiota and impaired gut barrier. Adiponectin as an anti-inflammatory adipokine may downregulate the deleterious immune response (the secretion of pro-inflammatory cytokines that are likely to modulate the pathogenesis of obesity and metabolic diseases), thus could prevent excessive weight gain in later life [58]. Findings from this study support the protective effect of breastfeeding on the future growth and development of infants. However, since the study only included the postnatal period up to 12 months, the effect of breastfeeding on infant growth trajectory beyond that age could not be finalized in the current study.

At 12 months of age, infants who were breastfed had a significantly lower body weight compared to infants who were not breastfed; however, the weight is still within the normal range. The duration of breastfeeding was shown to be related to decreased childhood overweight in a wide range of studies of weight status during the first year of age [60,61] or beyond [62,63,64,65,66]. The mechanisms regarding the effect of breastfeeding on the risk of overweight remain inconclusive. In contrast, several studies failed to establish a significant association between breastfeeding and childhood overweight [67,68,69,70,71]. A potential explanation for the discrepancy in the findings might be the difference in sample size, population age, proportion of breastfeeding, study design or diverse population (genetic and environmental background).

The presence of adiponectin in breast milk may be involved in the programming of energy balance in the early, critical period of infant growth and metabolic homeostasis development [72]. As a signaling molecule that regulates energy intake and expenditure, adiponectin is suggested to play a significant role in the regulation of appetite control in breastfed infants [73]. Short-term appetite control through breastfeeding on demand may influence infant metabolic programming, wherein it would enhance the infant’s ability to self-regulate nutrient intake [74,75]. Additionally, breastfed infants were shown to have greater satiety responsiveness compared to formula-fed infants [76], which subsequently may prevent excessive energy intake and lead to positive future weight trajectory [77]. 

Therefore, it is suggested that plausible underlying mechanisms for the protective effect of breastfeeding against adiposity may relate to the sufficient nutrients in breast milk and the presence of hormones and growth factors not found in infant formula [74]. Breastfed infants may absorb the right amount of nutrients according to the body’s needs, while unique bioactive compounds in breast milk may be involved in the inhibition of adipocyte differentiation [78]. Consequently, the optimal regulation or dysregulation of metabolic programming in early infancy may have an impact on the development of adiposity in later life.

### 4.3. Limitations and Strengths

This study did not measure maternal factors before conception. The findings are not comprehensive enough to provide data on mothers’ health before pregnancy, thus we are unable to explain the relationship between maternal pre-pregnancy health/nutritional status and infant adiposity, nor can we give insight into the intergenerational cycles of obesity.

Participant recruitment was conducted in a Southeast Asian setting with a Malaysian mother–offspring cohort comprised of the Malay population in a specific region. Therefore, generalizing the current findings to the national population should be done with caution.

The use of BAZ and abdominal circumference as surrogates for adiposity and adiposity changes during the first year of life were further limitations of this study. However, given that body weight and BMI are unable to differentiate between the depositions of fat mass and lean mass [79], the use of circumferential measure as an additional indicator may help provide a comprehensive assessment of adiposity [80].

A review reported that most studies suggested a 60% increased risk of obesity if rapid infant weight gain is observed before two years of age [81]. Since the duration of the study was only until the age of one year, data on infant weight change beyond that age could not be recorded. Hence, the findings from this study may only represent the tip of the iceberg of childhood obesity, which warrants further investigation. Nevertheless, the data are useful for giving an insight into the role of adipokines in the development of adiposity during the first year of age, since this is among the most critical periods in a human life.

The major strength of the present study is the application of a prospective cohort design that could be beneficial as baseline data for future pregnancy or birth cohort studies on infant adiposity, particularly in Malaysia. The measurements of maternal serum adiponectin and leptin levels throughout pregnancy, as well as the breast milk adiponectin level within two months postpartum, contribute to existing knowledge on adipokines. The data from this study demonstrated the effects of the studied adipokines on infant adiposity development. A better understanding of the relationship between these adipokines in mothers with infant adiposity development in the first year of life can be a significant finding to further research on the mechanism of infant adiposity through intrauterine programming.

## 5. Conclusions

In conclusion, maternal serum adiponectin during pregnancy and breast milk adiponectin were negatively associated with infant adiposity development in the first year of life. The positive findings regarding breast milk highlighted the beneficial nature of breastfeeding, which should be promoted to mothers of all races. In a clinical setting, the findings also underlined adiponectin as a potential biomarker and pharmacological treatment approach for obesity and its related diseases in the future. Keeping in mind that adiponectin is not routinely measured, future studies are warranted to verify which indicator—whether pre-pregnancy, early, mid- or late pregnancy weight status, or body fat—can be considered the best surrogate for adiponectin. Additionally, further human trials are needed in order to translate the observational research on the use of adiponectin as a clinical biomarker into therapeutic strategies in the future. 

## Figures and Tables

**Table 1:** Maternal characteristics ^a^.

Variable	*n* (%)	*p* *
Age, years	28.96 (4.52) ^b^	
Gestational age, weeks	16.24 (3.88) ^b^	
First trimester (≤13)	43 (27.7)	
Second trimester (14–24)	112 (72.3)	
Gravidity		
Primigravida	40 (25.8)	
Multigravida	39 (25.2)	
Grand multigravida	76 (49.0)	
Educational level		
Primary/secondary school	52 (33.5)	
Diploma	66 (42.6)	
Degree/Higher degree	37 (23.9)	
Employment status		
Employed	124 (80.0)	
Unemployed	31 (20.0)	
Monthly household income ^c^, RM		
<2300	62 (40.0)	
2300–5599	76 (49.0)	
≥5600	17 (11.0)	
Height, m	1.55 (0.06) ^b^	
Weight, kg		<0.001
Second trimester ^d^	57.53 (10.58) ^b^	
Third trimester	64.17 (10.61) ^b^	
Total gestational weight gain, kg	11.75 (5.42) ^b^	
Gestational weight gain rate, kg/week		<0.001
Second trimester	0.19 (0.22) ^b^	
Third trimester	0.28 (0.14) ^b^	
Pre-pregnancy weight, kg	54.41 (10.25) ^b^	
Pre-pregnancy BMI, kg/m^2^	22.58 (4.07) ^b^	
Pre-pregnancy BMI categories		
Underweight (<18.5 kg/m^2^)	23 (14.8)	
Normal (18.5–24.9 kg/m^2^)	93 (60.0)	
Overweight/Obese (≥25.0 kg/m^2^)	39 (25.2)	
Fasting blood glucose, mmol/L		0.745
Second trimester	4.81 (0.59) ^b^	
Third trimester	4.80 (0.54) ^b^	
Insulin, μU/mL		<0.001
Second trimester	5.88 (4.24) ^b^	
Third trimester	7.62 (8.16) ^b^	
Serum adiponectin, µg/mL (*n* = 151)		0.379
Second trimester	8.59 (6.54) ^b^	
Third trimester	7.64 (3.89) ^b^	
Serum leptin, ng/mL (*n* = 130)		0.019
Second trimester	38.31 (22.36) ^b^	
Third trimester	43.33 (21.45) ^b^	
Adiponectin to leptin ratio (ALR), µg/ng (*n* = 129)		0.321
Second trimester	0.38 (0.50) ^b^	
Third trimester	0.29 (0.43) ^b^	
Breast milk adiponectin, ng/mL (*n* = 135)		<0.001
0 month	17.05 (8.75) ^b^	
2 month	11.53 (8.45) ^b^	

Note: RM = Ringgit Malaysia; BMI = Body Mass Index. ^a^ *n* = 155, unless otherwise indicated; ^b^ Data were presented as mean (standard deviation); ^c^ Cut-off based on Tenth Malaysia Plan 2011–2015; ^d^ Measurements were taken either at recruitment or on the same day the blood samples were taken; * *p*-values based on paired sample *t*-test.

**Table 2:** Infant characteristics ^a^.

Variable	At Birth (*n* = 152)	2 Months (*n* = 151)	6 Months (*n* = 148)	12 Months (*n* = 147)	*p*
Weight, kg	3.10 (0.41) ^b,^*	5.15 (0.62)	7.13 (0.82)	8.56 (0.98)	<0.001 ^c^
BW categories (*n* (%))					
Low, <2.50 kg	10 (6.6)	-	-	-	
Normal, 2.50–4.00 kg	138 (90.8)	-	-	-	
High, >4.00 kg	4 (2.6)	-	-	-	
Length, cm	49.93 (2.66) ^d^	56.03 (2.25)	63.98 (2.24)	71.63 (2.36)	<0.001 ^c^
Ponderal Index, kg/m^3^	25.09 (3.75)	-	-	-	
Triceps skinfold, mm	-	-	8.20 (1.71)	7.57 (1.55)	
Subscapular skinfold, mm	-	-	6.81 (1.44)	6.43 (1.33)	
Abdominal circumference, cm	28.88 (2.49)	37.61 (2.39)	40.12 (2.61)	41.71 (2.86)	<0.001^ c^
Weight-for-age, *Z* scores	−0.50 (0.91)	−0.48 (0.82)	−0.68 (0.95)	−0.81 (0.95)	
Length-for-age, *Z* scores	−0.06 (1.53)	−1.02 (0.98)	−1.35 (0.97)	−1.42 (0.95)	
Body Mass Index (BMI), kg/m^2^	12.46 (1.53)	16.37 (1.38)	17.40 (1.48)	16.65 (1.41)	<0.001 ^c^
BMI-for-age, *Z* scores	−0.81 (1.30)	0.13 (0.91)	0.15 (0.97)	0.03(0.99)	
BMI-for-age categories (*n* (%))					
Severely wasted, *Z* < −3	8 (5.3)	0	0	0	
Wasted, *Z* < −2	20 (13.1)	1 (0.7)	1 (0.7)	2 (1.4)	
Normal, *Z* ≥ −2 to ≤1	117 (77.0)	125 (82.8)	117 (79.1)	124 (84.4)	
Risk of overweight, Z > 1	6 (3.9)	21 (13.9)	28 (18.9)	18 (12.2)	
Overweight, *Z* scores > 2	1 (0.7)	4 (2.6)	2 (1.3)	3 (2.0)	
Breastfeeding pattern (*n* (%))					0.002 ^f^
Exclusive	97 (63.8)	53 (35.1)	17 (11.5)	-	
Partial	55 (36.2)	93 (61.6)	93 (68.8)	79 (53.7)	
No	-	5 (3.3)	38 (25.7)	68 (46.3)	
Sex (*n* (%))					
Boy	68 (44.7)				
Girl	84 (55.3)				
Weaning age, month (*n* = 146)	*n *(%)				0.219 ^g^
<5.43 ^e^	50 (34.2)				
≥5.43	96 (65.8)				
6.00	69 (47.3)				

^a^ Data were presented as mean (standard deviation), unless otherwise indicated; ^b^ *p* = 0.003 vs. gestational age (Pearson’s correlation test); r = 0.24; ^c^ Values based on independent *t*-test between boy and girl infants at 2, 6 and 12 months of age; ^d^ *p* = 0.035 vs. gestational age (Pearson’s correlation test); r = 0.17; ^e^ Value based on mean of weaning age (standard deviation = 0.97); ^f^ Value based on independent *t*-test on infants weight between those who were breastfed and not breastfed at 12 months of age; ^g^ *p*-value based on Pearson’s correlation test (r = 0.10) with infants weight at 12 months of age; * *p* = 0.043 between boy and girl infants at birth (independent *t*-test).

**Table 3:** Regression coefficients (*p*-value) of maternal serum and breast milk adipokines on infant body weight.

Adipokines	Infant Body Weight (kg)							
	At Birth (*n* = 149)		2 Months (*n* = 136)		6 Months (*n* = 146)		12 Months (*n* = 142)	
	Simple Regression	Multiple Regression ^a^	Simple Regression	Multiple Regression ^b^	Simple Regression	Multiple Regression ^c^	Simple Regression	Multiple Regression ^c^
**Serum**								
*Second trimester*								
Adiponectin, µg/mL	−0.22 (0.023)	-	−0.29 (0.047)	-	−0.61 (0.001)	−0.59 (0.002)	−0.59 (0.010)	-
Leptin, ng/mL	0.01 (0.487)	-	0.02 (0.513)	-	0.02 (0.612)	-	0.07 (0.129)	-
ALR, µg/ng	−0.13 (0.052)	-	−0.17 (0.098)	-	−0.30 (0.027)	-	−0.41 (0.012)	-
*Third trimester*								
Adiponectin, µg/mL	−0.54 (0.002)	−0.47 (0.004)	−0.44 (0.097)	-	−1.05 (0.002)	-	−1.18 (0.005)	−1.02 (0.009)
Leptin, ng/mL	−0.03 (0.216)	-	−0.05 (0.081)	-	−0.04 (0.320)	-	−0.03 (0.578)	-
ALR, µg/ng	−0.07 (0.517)	-	−0.01 (0.922)	-	−0.18 (0.387)	-	−0.33 (0.179)	-
**Breast milk**								
*At birth*								
Adiponectin, ng/mL	-	-	−0.00 (0.985)	-	0.00 (0.947)	-	0.03 (0.741)	-
*2 months*								
Adiponectin, ng/mL	-	-	−0.61 (0.002)	−0.54 (0.003)	−0.30 (0.273)	-	0.02 (0.956)	-
**R^2^**, **%**	-	18.1	-	30.1	-	23.7	-	23.8

Note. ALR = Adiponectin to Leptin Ratio. ^a^ Adjusted for gestational weight gain, gestational age, infant sex and maternal age; ^b^ Adjusted for gestational weight gain, gestational age, infant sex and pre-pregnancy Body Mass Index; ^c^ Adjusted for gestational weight gain, gestational age, infant sex, pre-pregnancy Body Mass Index and breastfeeding pattern. Data were log-transformed before performing analysis.

**Table 4:** Regression coefficients (*p*-value) of maternal serum and breast milk adipokines on infant body mass index-for-age *Z* scores (BAZ).

Adipokines	Infant Body Mass Index-for-Age (Z-Scores)							
	At Birth (n = 152)		2 Months (n = 135)		6 Months (n = 145)		12 Months (n = 142)	
	Simple Regression	Multiple Regression a	Simple Regression	Multiple Regression b	Simple Regression	Multiple Regression b	Simple Regression	Multiple Regression c
**Serum**								
*Second trimester*								
Adiponectin, µg/mL	−0.82 (0.009)	-	−0.43 (0.040)	-	−0.58 (0.012)	−0.62 (0.009)	−0.48 (0.038)	-
Leptin, ng/mL	0.06 (0.316)	-	−0.79 (0.006)	-	0.01 (0.846)	-	0.05 (0.335)	-
ALR, µg/ng	−0.53 (0.016)	−0.94 (0.000)	−0.22 (0.140)	-	−0.26 (0.108)	-	−0.29 (0.079)	-
*Third trimester*								
Adiponectin, µg/mL	−1.64 (0.003)	-	−0.43 (0.258)	-	−0.76 (0.067)	-	−0.99 (0.021)	−1.00 (0.018)
Leptin, ng/mL	−0.09 (0.178)	−0.22 (0.001)	−0.07 (0.115)	-	−0.03 (0.486)	-	−0.05 (0.329)	-
ALR, µg/ng	−0.18 (0.570)	-	0.09 (0.693)	-	−0.07 (0.773)	-	−0.18 (0.490)	-
**Breast milk**								
*At birth*								
Adiponectin, ng/mL	-	-	0.06 (0.364)	-	0.04 (0.585)	-	0.06 (0.453)	-
*2 months*								
Adiponectin, ng/mL	-	-	−0.79 (0.006)	−0.79 (0.008)	−0.06 (0.861)	-	−0.02 (0.946)	-
**R^2^**, **%**	-	21.1	-	6.3	-	6.0	-	9.1

Note. ALR = Adiponectin to Leptin Ratio. ^a^ Adjusted for gestational weight gain, gestational age, infant sex and maternal age; ^b^ Adjusted for gestational weight gain and infant sex; ^c^ Adjusted for gestational weight gain, infant sex and breastfeeding pattern. Data were log-transformed before performing analysis.

**Table 5:** Regression coefficients (*p*-value) of maternal serum and breast milk adipokines on infant abdominal circumference.

Adipokines	Infant abdominal circumference (cm)							
	At Birth (*n* = 146)		2 Months (*n* = 136)		6 Months (*n* = 146)		12 Months (*n* = 131)	
	Simple Regression	Multiple Regression ^a^	Simple Regression	Multiple Regression ^b^	Simple Regression	Multiple Regression ^b^	Simple Regression	Multiple Regression
**Serum**								
*Second trimester*								
Adiponectin, µg/mL	−1.20 (0.026)	-	−1.34 (0.017)	-	−1.38 (0.024)	−1.42 (0.023)	−0.59 (0.010)	-
Leptin, ng/mL	−0.03 (0.802)	-	0.03 (0.817)	-	0.04 (0.745)	-	0.07 (0.129)	-
ALR, µg/ng	−0.49 (0.206)	-	−0.71 (0.070)	-	−0.70 (0.100)	-	−0.85 (0.076)	-
*Third trimester*								
Adiponectin, µg/mL	−2.66 (0.005)	−2.52 (0.009)	−1.76 (0.083)	-	−2.12 (0.058)	-	−1.18 (0.005)	-
Leptin, ng/mL	−0.13 (0.271)	-	−0.21 (0.089)	-	−0.19 (0.162)	-	−0.03 (0.578)	-
ALR, µg/ng	−0.19 (0.731)	-	−0.08 (0.884)	-	−0.00 (0.999)	-	−0.21 (0.772)	-
**Breast milk**								
*At birth*								
Adiponectin, ng/mL	-	-	−1.12 (0.497)	-	−0.09 (0.640)	-	0.03 (0.741)	-
*2 months*								
Adiponectin, ng/mL	-	-	−2.49 (0.001)	−2.34 (0.003)	−1.01 (0.251)	-	0.02 (0.956)	-
**R^2^**, **%**	-	8.8	-	16.4	-	10.2	-	-

Note. ALR = Adiponectin to Leptin Ratio. ^a^ Adjusted for gestational weight gain, gestational age, infant sex and maternal age; ^b^ Adjusted for gestational weight gain, gestational age, infant sex and breastfeeding pattern. Data were log-transformed before performing analysis.
